# A Retrospective Cohort Study on the Association between Red Cell Distribution Width and All-Cause Mortality of Patients with Cholecystitis at ICU Admission

**DOI:** 10.1155/2021/9625220

**Published:** 2021-10-13

**Authors:** Yihua Dong, Yu Pan, Wei Zhou, Yanhuo Xia, Jingye Pan

**Affiliations:** ^1^Department of Intensive Care Unit, The First Affiliated Hospital of Wenzhou Medical University, Wenzhou, Zhejiang 325000, China; ^2^Department of Pharmacy, Wenzhou Hospital of Integrated Traditional Chinese and Western Medicine, Wenzhou, Zhejiang 325000, China

## Abstract

**Background:**

Elevated red cell distribution width (RDW) has been reported to be associated with mortality in some critically ill patient populations. The aim of this article is to investigate the relationship between RDW and in-hospital mortality and short- and long-term mortality of patients with cholecystitis.

**Method:**

We conducted a retrospective cohort study in which data from all 702 patients extracted from the Medical Information Mart for Intensive Care III (MIMIC-III) database were used. Receiver operating characteristic (ROC) curves were constructed to evaluate the prognostic predictive value of RDW for in-hospital mortality and short- (i.e., 30-day and 90-day) and long-term (i.e., 180-day, 1-year, 3-year, and 5-year) mortality. We converted RDW into a categorical variable according to quintiles as less than or equal to 13.9%, 14.0-14.8%, 14.9-15.8%, and 15.9-17.2% and more than 17.2%. The Kaplan-Meier (K-M) methods and log-rank tests were used to compare survival differences among different groups. The relationships between RDW levels and in-hospital mortality were evaluated by univariate and multivariate binary logistic regression models. Multivariable Cox regression models were built to investigate the association of RDW on the short-term and long-term mortality.

**Result:**

After adjusting for potential confounders, RDW was positively associated with in-hospital mortality (OR: 1.187, 95% CI [1.049, 1.343]) and short- (i.e., 30-day: HR: 1.183, 95% CI [1.080, 1.295], 90-day: HR: 1.175, 95% CI [1.089, 1.268]) and long-term (i.e., 1-year: HR:1.162, 95% CI [1.089, 1.240]) mortality in critically ill patients with cholecystitis. Similar results were also shown in the secondary outcomes of 180-day, 3-year, and 5-year mortality. RDW had a significant accurate prognostic effect on different endpoints and could improve the prognostic effect of scoring systems.

**Conclusion:**

High level of RDW is associated with an increased risk of in-hospital mortality and short- and long-term mortality in critically ill patients with cholecystitis. RDW can independently predict the prognosis of patients with cholecystitis.

## 1. Introduction

Cholecystitis is inflammation of the gallbladder, due to the presence of gallstones or bacterial infection. It is the sixth most common disease in emergency departments [1] and also common in intensive care units. As we know that the presence of gallstones is the most common cause, about 85% of acute cholecystitis is attributed to gallstones, and the morbidity is approximately 10-15% [2]. In addition, about 15% of cholecystitis has no evidence of calculi. In the critically ill patients, it is reported that about 50% of acute cholecystitis is acalculous [3]. Whatever the cause, the acute inflammation of the gallbladder may lead to poor prognosis. It is reported that the mortality of acute cholecystitis is 5% to 10% [4]. Red blood cell distribution width (RDW) is a commonly used parameter to measure the variability of red blood cell size [5]. It is cheap and convenient to obtain, and it has been shown to be associated with many disorders. RDW as a valuable predictor of clinical prognosis has been documented in patients with cardiovascular [6], kidney [7], lung [8], and liver [9] diseases and those undergoing cancer [10] and sepsis [11]. And it has been considered to be associated with inflammation in more recent years [12]. Moreover, RDW has been shown to predict all-cause mortality in critically ill patients [13].

Inflammation plays an important role in the progression for cholecystitis. And several inflammation markers can predict mortality of cholecystitis which has been confirmed. However, the relationship between RDW and prognosis of patients with cholecystitis has not been established. Therefore, in our study, the aim was to investigate whether RDW is independently related to in-hospital mortality and short- and long-term mortality and if there is an association between admission RDW and all-cause mortality of ICU patients with cholecystitis.

## 2. Methods

### 2.1. Data Source

We performed a retrospective cohort study using data searched from the Medical Information Mart for Intensive Care III (MIMIC-III) [14], a publicly available critical care database developed by the Computational Physiology Laboratory of Massachusetts Institute of Technology (MIT). This extensive ICU clinical database consists of deidentified data from ICU patients who received care at the Beth Israel Deaconess Medical Center (BIDMC) from 2001 to 2012, including demographic characteristics, vital signs, and laboratory tests. One of the authors of our study (Y.H. Dong, certification number: 22691479) obtained access to the database after online training at the National Institutes of Health (NIH). The institutional review boards of MIT and BIDMC approved to access the database. Since this was a retrospective cohort study, there was no need for informed consent.

### 2.2. Population Selection Criteria

**Figure 1:**
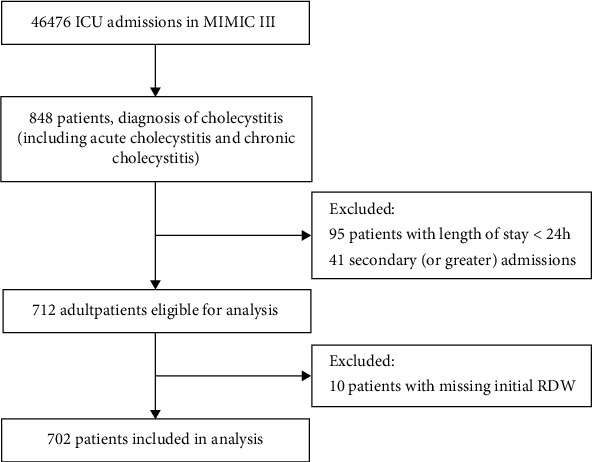
Flowchart of the study.

A total of 46,476 ICU patients were recruited to BIDMC between 2001 and 2012. We retrospectively analyzed adult patients (≥18 years) who were diagnosed with cholecystitis according to the Ninth Revision of International Classification of Diseases (ICD-9) code at the first ICU admission. 95 patients stayed in the ICU for less than 24 hours, and 41 secondary (or more) admission patients were excluded. We also excluded patients without admission RDW (10 patients). Finally, the cohort included 702 patients (Figure 1).

### 2.3. Data Extraction

From the MIMIC-III database, we used structured query language (SQL) with Navicat Premium software (version 12.0.29) to extract the following information: demographic information, laboratory parameters, scoring systems, and major comorbidities. Laboratory parameters included RDW, white blood count (WBC), red blood cell (RBC), blood urea nitrogen (BUN), hematocrit, creatinine, mean corpuscular volume (MCV), and mean corpuscular hemoglobin (MCH) at baseline. Scoring systems were recorded, including Sequential Organ Failure Assessment (SOFA) score, Logistic Organ Dysfunction System (LODS), Simplified Acute Physiology Score II (SAPSII), Quick Sepsis-Related Organ Failure Assessment (QSOFA) score, and Systemic Inflammatory Response Syndrome (SIRS) score. During the first 24 h of ICU admission, all variables were recorded. All the above scores were calculated with clinical information, and the comorbidities were estimated by the Elixhauser Comorbidity Index (State Inpatient Database (SID) 30). RDW within 24 hours of admission was the exposure of interest, and we converted it into a categorical variable according to quintiles as less than or equal to 13.9%, 14.0-14.8%, 14.9-15.8%, and 15.9-17.2% and more than 17.2%.

### 2.4. Outcomes

The primary outcomes in our study were all-cause in-hospital mortality, 30-day mortality, 90-day mortality, and 1-year mortality. The date of admission was the starting date of follow-up, and the date of death was obtained from the Social Security database.

### 2.5. Statistical Analysis

Categorical variables were shown as numbers and percentages, and continuous variables were summarized as median and interquartile range (IQR). The chi-square (or Fisher's test) and Kruskal-Wallis test was used for comparison of categorical and continuous variables between groups.

The relationships between RDW levels and in-hospital mortality were evaluated by univariate and multivariable binary logistic regression models. The Hosmer-Lemeshow test was used to evaluate the suitability of the model. Multivariable Cox regression models were built to analyze the association of RDW levels on short- (i.e., 30-day and 90-day) and long-term (i.e., 1-year) mortality. The Kaplan-Meier (K-M) methods and log-rank tests were used to compare survival differences among different groups. These results were expressed as odd ratios (ORs) with 95% CIs or hazard ratios (HRs) with 95% CIs. Potentially significant (*P* < 0.1) confounders or clinically important predictors mentioned in the past literature were considered in multivariable logistic/Cox regression models. To ensure the robustness of our analysis, a sensitivity analysis was conducted. Prognostic performance of RDW, SOFA, LODS, QSOFA, SAPSII, SIRS, and their combination for in-hospital mortality and short- and long-mortality in critically ill patients with cholecystitis was evaluated by using the receiver operating characteristic (ROC) curve. ROC curve analysis statistics were evaluated using the area under the curve (AUC). The coefficients (*β*) of each variable were obtained by the logistic regression model. We calculated a new variable *Y* according to the equation: *Y* = exp (*β*_0_ + *β*_1_*X*_1_ + *β*_2_*X*_2_+⋯+*β*_*n*_*X*_*n*_)/1 + exp (*β*_0_ + *β*_1_*X*_1_ + *β*_2_*X*_2_+⋯+*β*_*n*_*X*_*n*_) [12], where *X* represents each included variables and in the present situation it refers to RDW, SOFA, LODS, QSOFA, SAPSII, and SIRS. *Y* is a new parameter calculated by combining all variables such as RDW, SOFA, LODS, QSOFA, SAPSII, and SIRS in the regression model. In addition, we divided subgroup patients into those with and without sepsis and compared the prognostic effect on different endpoints.

A stratification analysis by age, ethnicity, first care unit, scoring systems, sepsis, comorbidities, and so on was performed to examine the association of RDW and 1-year mortality between subgroups. The significance of the interaction was tested to estimate the interaction effect by the likelihood ratio test. All analyses were performed using SPSS 23, MedCalc (version 20.0.11), and statistical package R (version 4.0.5). A two-tailed *P* value < 0.05 was considered to indicate statistical significance.

## 3. Results

### 3.1. Baseline Characteristics and Follow-Up

We enrolled and collected samples from 702 patients, and the clinical characteristics are summarized in Table 1. Among them, 398 were male and 304 were female, with a median age of 70.00 years (IQR 58.00-80.00). The majority of the patients were White (76.2%). The median admission RDW was 15.3% and ranged from 14.2% to 16.8%. Hypertension (58.1%), cardiac arrhythmias (40.5%), and congestive heart failure (30.9%) were the three most common comorbidities. In general, in-hospital mortality was 15.0% with 597 survivors and 105 nonsurvivors. Postdischarge mortality rates were 13.8% at 30 days, 21.7% at 90 days, and 30.2% at 1 year.

We categorized patients of the study cohort in five identical groups, according to RDW levels on admission. There were statistically significant differences between stratified groups in age, SOFA, LODS, SAPSII, QSOFA, survival days, and comorbidities (*P* < 0.05), and statistically significant differences were found on in-hospital mortality and 30-day, 90-day, and 1-year mortality. Patients with higher RDW had higher long-term mortality, as well as the short-term mortality (*P* < 0.001 for all).

### 3.2. Survival Status of Patients with Different RDW Levels

**Figure 2:**
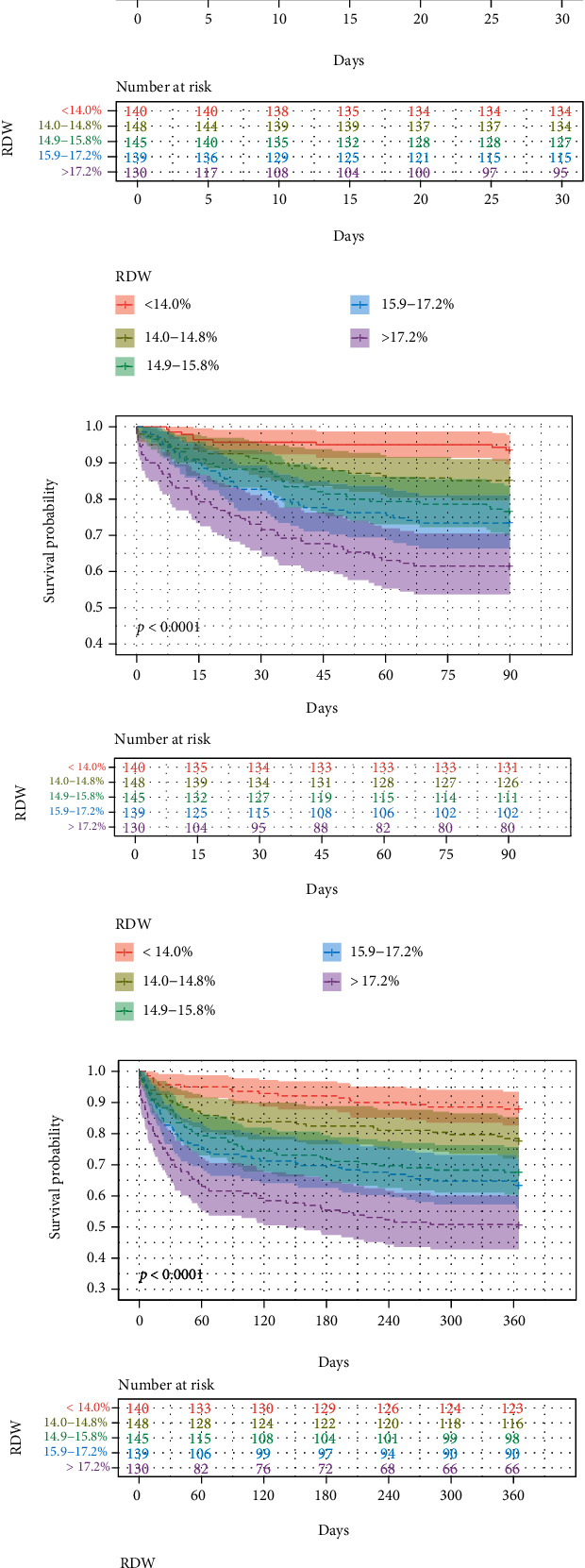
Overall survival of different RDW groups. Kaplan-Meier curve of 30-day (a), 90-day (b), and 1-year (c) mortality.

We plotted Kaplan-Meier survival curves and compared the prognosis between different groups by the log-rank test. The 30-day, 90-day, and 1-year survival was significantly lower in the high-RDW group (30-day survival: 95.70% versus 90.50% versus 87.60% versus 82.70% versus 73.10%; 90-day survival: 93.60% versus 85.10% versus 76.60% versus 73.40% versus 61.50%; 1-year survival: 87.90% versus 77.70% versus 67.60% versus 63.30% versus 50.80%, all *P* < 0.001) (Figure 2). Similarly, there were significant differences in 180-day, 3-year, and 5-year survival rates among different RDW levels on admission (Figure S1). The high-RDW group had a poor short- (i.e., 30-day and 90-day) and long-term (i.e., 180-day, 1-year, 3-year, and 5-year) prognosis in critically ill patients with cholecystitis (Table S1).

### 3.3. ROC Curve Analysis

**Figure 3:**
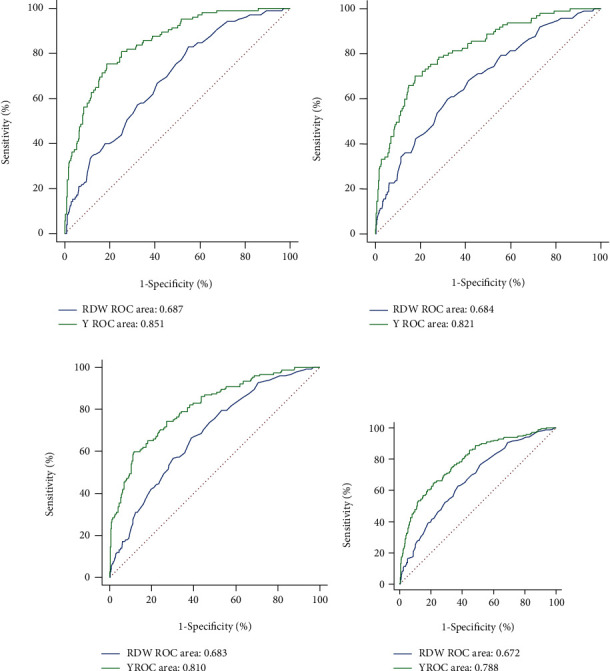
ROC curves showing the diagnostic performance of admission RDW in predicting in-hospital mortality (a) and 30-day (b), 90-day (c), and 1-year (d) mortality.

Table 2 lists the predictive values of RDW and severity scoring systems (i.e., LODS, SAPSII, SOFA, QSOFA, and SIRS) for in-hospital mortality and 30-day, 90-day, and 1-year mortality. In terms of 1-year mortality, the area under the ROC curve (AUROC) of RDW, SAPSII, LODS, SOFA, QSOFA, and SIRS was 0.672 (95% CI [0.636, 0.707]), 0.763 (95% CI [0.730, 0.794]), 0.730 (95% CI [0.695, 0.762]), 0.671 (95% CI [0.635, 0.706]), 0.572 (95% CI [0.534, 0.608]), and 0.546 (95% CI [0.508, 0.583]), respectively. In addition, we performed ROC curve analysis to assess discrimination abilities of RDW compared with severity scoring systems for 180-day, 3-year, and 5-year mortality (Table S2). The predictive value of RDW for both long- and short-term mortality was significantly accurate and significantly higher than that of QSOFA (1-year: 95% CI [0.534, 0.608], AUROC = 0.572, *P* = 0.0003) and SIRS (1-year: 95% CI [0.508, 0.583], AUROC = 0.546, *P* < 0.0001). Compared with LODS, SAPSII, SOFA, QSOFA, or SIRS alone, the AUC of each severity score combined with RDW was significantly higher in predicting long-term mortality (i.e., 180-day, 1-year, 3-year, and 5-year) in patients with cholecystitis (*P* < 0.05 for all). Similar results were found in predicting in-hospital mortality and short-term mortality (i.e., 30-day and 90-day) in patients with cholecystitis (Table 2). RDW may improve the prognostic efficiency of scoring systems such as LODS, SAPSII, SOFA, QSOFA, and SIRS. Admission RDW had moderate discriminating power, and the combination of risk scores significantly improved the diagnostic performance (i.e., 1-year: 0.788, 95% CI [0.756, 0.818]) (Figure 3). The predictive value of RDW to predict different clinical endpoints was significantly better in the sepsis group than in the nonsepsis group (i.e., 1-year: 0.698, 95% CI [0.647, 0.748] vs. 0.591, 95% CI [0.511, 0.671], *P* < 0.001) (Table 3).

### 3.4. Association between RDW Levels and Outcomes

In our study, we developed three models (univariate and multivariate binary logistic regression and Cox hazard regression) to examine the independent predictive value of RDW for in-hospital and short- and long-term mortality in patients with cholecystitis. We adjusted potential confounders in three models. The adjusted OR (95% CI) for RDW was 1.187 (1.049, 1.343) (*P* = 0.007) for in-hospital mortality of the patients. The adjusted HRs (95% CI) for RDW were 1.183 (1.080, 1.295), 1.175 (1.089, 1.268), and 1.162 (1.089, 1.240) for 30-day, 90-day, and 1-year mortality, respectively (*P* < 0.001 for all). Then, we converted the continuous variable of RDW to a categorical variable for regression analysis. The logistic/Cox regression analysis, in which patients with the lowest level of RDW (<14.0%) were used as the reference group, was used to assess whether increased RDW level was associated with different endpoints. In terms of 1-year mortality, the adjusted HRs (95% CI) were 1.982 (1.081, 3.637), 2.557 (1.435, 4.556), 2.558 (1.444, 4.531), and 3.208 (1.812, 5.679). We found that the trend of the effect size in different RDW groups was equidistant, which was consistent with *P* for trend of RDW with in-hospital mortality (Table 4) and short- and long-term mortality (Table 5 and Table S3). In-hospital mortality showed similar trends; the higher the RDW level, the greater the risk of in-hospital mortality. The lower RDW level (14.0-14.8%, *P* = 0.117) led to a slightly increased risk of in-hospital mortality (Table 4). After adjusting for potential confounders, high level of RDW was significantly associated with an increased risk of in-hospital mortality and short- (i.e., 30-day and 90-day) and long-term (i.e., 180-day, 1-year, 3-year, and 5-year) mortality (*P* < 0.05 for all).

### 3.5. Subgroup Analyses

**Figure 4:**
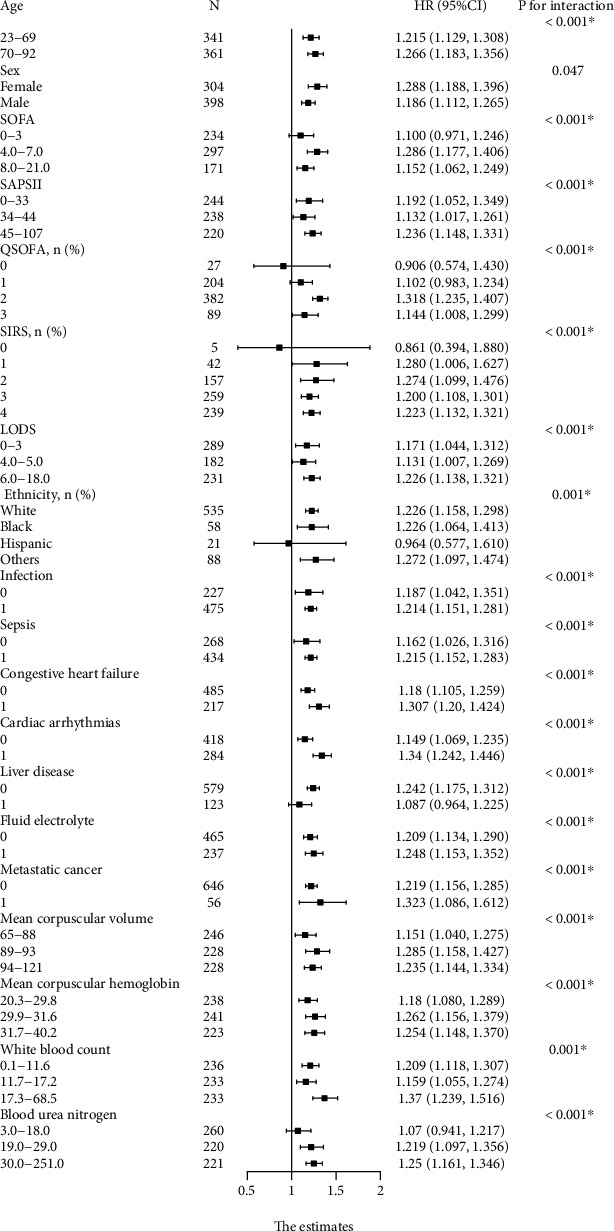
Subgroup analysis of the association between RDW and 1-year mortality.

We performed stratification analyses of 1-year mortality by age, gender, ethnicity, first care unit, admission type, scoring systems, laboratory parameters, sepsis, and comorbidities to observe the trend of effect sizes in these variables between subgroups. The relationship between each subgroup and RDW is shown in Figure 4. RDW of patients less than 70 years old was associated with high risks of 1-year mortality (HR: 1.215, 95% CI [1.129, 1.308], *P* < 0.001). Older subjects were also observed to have a higher risk (HR: 1.266, 95% CI [1.183, 1.356], *P* < 0.001). We converted continuous variables (i.e., SOFA, SAPSII, and LODS) into categorical variables, which were stratified into terciles. In SAPSII and LODS subgroups, there were U-shaped relationships between RDW and 1-year mortality, while in SOFA and QSOFA subgroups, there were inverted U-shaped relationships. There were significant changes in MCV, MCH, WBC, BUN, congestive heart failure, cardiac arrhythmias, and metastatic cancer (*P* < 0.05 for all) (Figure 4). RDW of sepsis patients was associated with high risks of 1-year mortality (HR: 1.215, 95% CI [1.152, 1.283], *P* < 0.001). However, liver disease was found to have the opposite effect, with the RDW in patients without liver disease having a higher risk of 1-year mortality (HR: 1.242, 95% CI [1.175, 1.312], *P* < 0.001). In addition, no association between RDW and 1-year mortality was found in the subgroups of hypertension, diabetes, blood loss anemia, and deficiency anemias (*P* > 0.05) (Table S4).

## 4. Discussion

The purpose of this study was to investigate the relationship between RDW and all-cause mortality in patients with cholecystitis in the intensive care units. Results of the study indicated that after adjusting for potential confounders, high level of RDW was associated with an increased risk of in-hospital mortality and short- (i.e., 30-day and 90-day) and long-term (i.e., 180-day, 1-year, 3-year, and 5-year) mortality in critically ill patients with cholecystitis. RDW was an independent prognostic indicator of different endpoints. RDW had a significant accurate prognostic effect on different endpoints and could improve the prognostic effect of scoring systems such as LODS, SAPSII, SOFA, QSOFA, and SIRS. The combination of RDW and risk scores significantly improved the diagnostic performance. Therefore, RDW may be a useful predictor of mortality in patients with cholecystitis admitted to the intensive care units. The predictive value of RDW to predict different clinical endpoints was significantly better in the sepsis group than in the nonsepsis group.

Previous studies have shown the association of higher RDW and poor survival outcomes in acute kidney injury [7], idiopathic pulmonary fibrosis [8], sepsis [15], infective endocarditis [16], and so on. Our study is to analyze the predictive value of RDW in in-hospital mortality and short - and long-term mortality in critically ill patients with cholecystitis.

The mechanism that causes this relationship is not clear; some previous studies have suggested several plausible explanations. First, severe inflammation has been shown to inhibit bone marrow hyperplasia, reduce iron bioavailability, promote erythropoietin resistance, and increase erythrocyte apoptosis [17–19]. These changes by inflammation lead to anemia, and the lifetime of large and small red blood cells is extended by changing the clearance standard of red blood cells by size [20], eventually leading to an increase in RDW. Second, oxidative stress is another possible factor of the association between RDW and mortality in patients with cholecystitis. Reactive oxygen species can promote the expression of proinflammatory mediators [21], and high levels of oxidative stress can decrease the survival of red blood cells and enhance the release of reticulocytes into the peripheral [22]. In addition, there are several factors that cannot be ignored. Malnutrition is common in patients with cholecystitis and is known to increase RDW. Among all patients admitted to the intensive care units, endothelial dysfunction is common, which can also cause an increase in RDW [23]. Usual ultrasound or computed tomography can confirm the diagnosis of cholecystitis, but these diagnostic tools are not sensitive enough to evaluate the severity and prognosis of the disease [24].

There are some biomarkers that can predict the severity of cholecystitis, such as WBC, C-reactive protein (CRP), and procalcitonin (PCT). In the TG 18, WBC is the important criterion for assessing the severity of acute cholecystitis [25]. But in our study, the predictive power of WBC was much lower than that of RDW. In addition, the SOFA score is an important means to assess disease severity and expected mortality in the intensive care units. It has been reported that RDW has a stronger prognostic ability than that of the SOFA score in sepsis shock patients [26]. We found that RDW can improve the prognostic efficiency of scoring systems such as LODS, SAPSII, SOFA, QSOFA, and SIRS.

There are several limitations of the study. First, we did not investigate blood transfusions, use of erythropoietin, iron storage status, and other nutritional deficiencies, which may affect RDW. Second, the study population comprised heterogeneous subphenotypes, and the predictive performance of RDW can differ across subgroups [27]. Finally, we also do not have data on specific causes of death, so we cannot examine other causes of death. Therefore, in order to further clarify the predictive value of RDW, more rigorous and prospective studies are needed.

## 5. Conclusions

RDW is a simple and cost-effective laboratory test, and our analysis indicates that, after adjusting for potential confounders, high level of RDW was associated with an increased risk of in-hospital mortality and short- and long-term mortality in patients admitted to the ICU with cholecystitis. RDW was an independent prognostic indicator of different endpoints. This association may enable RDW to be used as a prognostic indicator to provide useful information for the analysis of early risk grading of cholecystitis in critically ill patients. In addition, RDW can improve the prognostic efficiency of scoring systems such as LODS, SAPSII, SOFA, QSOFA, and SIRS. Therefore, RDW can be used as a reference to evaluate the prognosis of ICU patients with cholecystitis. Further studies are needed to reveal the explicit mechanism and to elucidate the dynamic changes of RDW in the progression of cholecystitis for different endpoints.

## Figures and Tables

**Table 1:** Baseline characteristics and clinical outcomes of all patients.

Variable	All patients (*n* = 702)	Discharge red cell distribution width					*P* value
		≤13.9%	14.0-14.8%	14.9-15.8%	15.9-17.2%	≥17.3%	
		(*n* = 140)	(*n* = 148)	(*n* = 145)	(*n* = 139)	(*n* = 130)	
Age (years)	70.00 (58.00-80.00)	69.50 (57.00-81.00)	71.00 (60.25-81.00)	73.00 (62.50-83.50)	68.00 (57.00-78.00)	66.00 (53.50-78.25)	0.015^∗^
Sex (male), *n* (%)	398 (56.7%)	74 (52.9%)	78 (52.7%)	84 (57.9%)	83 (59.7%)	79 (60.8%)	0.511
Time in ICU (days)	2.92 (1.61-6.69)	2.23 (1.13-4.36)	2.76 (1.63-6.96)	2.92 (1.79-7.38)	3.39 (1.90-7.86)	3.79 (1.77-7.80)	0.002^∗^
Time in hospital (days)	11.81 (7.14-21.37)	8.37 (5.83-15.84)	9.90 (6.83-17.52)	12.31 (7.83-23.54)	14.40 (8.34-24.07)	15.88 (8.15-28.71)	<0.001^∗^
Scoring systems							
SOFA	5.00 (3.00-7.00)	4.00 (2.00-5.75)	4.00 (3.00-6.00)	5.00 (3.00-7.00)	5.00 (3.00-8.00)	7.00 (4.00-10.00)	<0.001^∗^
LODS	4.00 (2.00-6.00)	3.00 (2.00-4.00)	3.50 (2.00-6.00)	4.00 (3.00-7.00)	4.00 (3.00-7.00)	5.50 (3.00-8.00)	<0.001^∗^
SAPSII	38.00 (30.00-47.25)	34.00 (26.00-40.00)	37.00 (28.25-47.00)	40.00 (32.00-48.00)	40.00 (31.00-50.00)	42.50 (33.75-55.00)	<0.001^∗^
QSOFA, *n* (%)							0.008^∗^
0	27 (3.8%)	6 (4.3%)	8 (5.4%)	3 (2.1%)	5 (3.6%)	5 (3.8%)	
1	204 (29.1%)	54 (38.6%)	31 (20.9%)	43 (29.7%)	41 (29.5%)	35 (26.9%)	
2	382 (54.4%)	74 (52.9%)	87 (58.8%)	81 (55.9%)	77 (55.4%)	63 (48.5%)	
3	89 (12.7%)	6 (4.3%)	22 (14.9%)	18 (12.4%)	16 (11.5%)	27 (20.8%)	
SIRS, *n* (%)							0.206
0	5 (0.7%)	1 (0.7%)	2 (1.4%)	1 (0.7%)	0 (0.0%)	1 (0.8%)	
1	42 (6.0%)	12 (8.6%)	7 (4.7%)	5 (3.4%)	13 (9.4%)	5 (3.8%)	
2	157 (22.4%)	37 (26.4%)	30 (20.3%)	33 (22.8%)	27 (19.4%)	30 (23.1%)	
3	259 (36.9%)	51 (36.4%)	58 (39.2%)	61 (42.1%)	52 (37.4%)	37 (28.5%)	
4	239 (34.0%)	39 (27.9%)	51 (34.5%)	45 (31.0%)	47 (33.8%)	57 (43.8%)	
Ethnicity, *n* (%)							0.164
White	535 (76.2%)	105 (75.0%)	116 (78.4%)	116 (80.0%)	96 (69.1%)	102 (78.5%)	
Black	58 (8.3%)	8 (5.7%)	8 (5.4%)	12 (8.3%)	16 (11.5%)	14 (10.8%)	
Hispanic	21 (3.0%)	5 (3.6%)	4 (2.7%)	1 (0.7%)	8 (5.8%)	3 (2.3%)	
Others	88 (12.5%)	22 (15.7%)	20 (13.5%)	16 (11.0%)	19 (13.7%)	11 (8.5%)	
First care unit, *n* (%)							0.672
MICU	259 (36.9%)	49 (35.0%)	50 (33.8%)	53 (36.6%)	48 (34.5%)	59 (45.4%)	
CCU	36 (5.10%)	10 (7.1%)	5 (3.4%)	5 (3.4%)	10 (7.2%)	6 (4.6%)	
SICU	257 (36.6%)	47 (33.6%)	61 (41.2%)	53 (36.6%)	55 (39.6%)	41 (31.5%)	
CSRU	56 (8.0%)	11 (7.9%)	14 (9.5%)	14 (9.7%)	9 (6.5%)	8 (6.2%)	
TSICU	94 (13.4%)	23 (16.4%)	18 (12.2%)	20 (13.8%)	17 (12.2%)	16 (12.3%)	
Admission type, *n* (%)							0.273
Emergency	544 (77.5%)	106 (75.7%)	108 (73.0%)	112 (77.2%)	111 (79.9%)	107 (82.3%)	
Elective	137 (19.5%)	28 (20.0%)	36 (24.3%)	29 (20.0%)	27 (19.4%)	17 (13.1%)	
Urgent	21 (3.00%)	6 (4.3%)	4 (2.7%)	4 (2.8%)	1 (0.7%)	6 (4.6%)	
Major comorbidities, *n* (%)							
Congestive heart failure	217 (30.9%)	31 (22.1%)	37 (25.0%)	50 (34.5%)	51 (36.7%)	48 (36.9%)	0.013^∗^
Cardiac arrhythmias	284 (40.5%)	52 (37.1%)	58 (39.2%)	59 (40.7%)	60 (43.2%)	55 (42.3%)	0.853
Hypertension	408 (58.1%)	86 (61.4%)	89 (60.1%)	84 (57.9%)	75 (54.0%)	74 (56.9%)	0.746
Chronic pulmonary	155 (22.1%)	25 (17.9%)	34 (23.0%)	36 (24.8%)	31 (22.3%)	29 (22.3%)	0.706
Renal failure	120 (17.1%)	17 (12.1%)	13 (8.8%)	26 (17.9%)	33 (23.7%)	31 (23.8%)	0.001^∗^
Liver disease	123 (17.5%)	11 (7.9%)	12 (8.1%)	32 (22.1%)	31 (22.3%)	37 (28.5%)	<0.001^∗^
Metastatic cancer	56 (8.0%)	4 (2.9%)	14 (9.5%)	8 (5.5%)	15 (10.8%)	15 (11.5%)	0.034^∗^
Solid tumor	22 (3.1%)	0 (0.0%)	5 (3.4%)	5 (3.4%)	7 (5.0%)	5 (3.8%)	0.166
Coagulopathy	134 (19.1%)	13 (9.3%)	19 (12.8%)	27 (18.6%)	30 (21.6%)	45 (34.6%)	<0.001^∗^
Sepsis	434 (61.8%)	75 (53.6%)	79 (53.4%)	97 (66.9%)	93 (66.9%)	90 (69.2%)	0.006^∗^
Survive days	133.34 (21.10-649.51)	419.90 (108.59-1389.17)	321.38 (35.57-1057.06)	100.34 (31.07-547.73)	102.71 (20.35-535.49)	48.66 (9.22-388.29)	<0.001^∗^
Mortality rates, *n* (%)							
In-hospital	105 (15.0%)	5 (3.6%)	13 (8.8%)	26 (17.9%)	23 (16.5%)	38 (29.2%)	<0.001^∗^
30-day	97 (13.8%)	6 (4.3%)	14 (9.5%)	18 (12.4%)	24 (17.3%)	35 (26.9%)	<0.001^∗^
90-day	152 (21.7%)	9 (6.4%)	22 (14.9%)	34 (23.4%)	37 (26.6%)	50 (38.5%)	<0.001^∗^
180-day	178 (25.4%)	11 (7.9%)	26 (17.6%)	41 (28.3%)	42 (30.2%)	58 (44.6%)	<0.001^∗^
1-year	212 (30.2%)	17 (12.1%)	33 (22.3%)	47 (32.4%)	51 (36.7%)	64 (49.2%)	<0.001^∗^

^∗^ *P* < 0.05.

**Table 2:** Area under the receiver operating curve of RDW and scoring systems for predicting in-hospital, 30-day, 90-day, and 1-year mortality.

	Hospital mortality		30-day mortality		90-day mortality		1-year mortality	
	AUC (95% CI)	*P*	AUC (95% CI)	*P*	AUC (95% CI)	*P*	AUC (95% CI)	*P*
RDW	0.687 (0.651-0.721)		0.684 (0.648-0.718)		0.683 (0.647-0.717)		0.672 (0.636-0.707)	
LODS	0.822 (0.792-0.850)	*P* = 0.0455	0.789 (0.757-0.818)	*P* = 0.0246	0.768 (0.735-0.798)	*P* = 0.0065	0.730 (0.695-0.762)	*P* = 0.0003
LODS∗RDW	0.833 (0.803-0.860)		0.801 (0.769-0.830)		0.779 (0.746-0.809)		0.744 (0.710-0.776)	
SAPSII	0.817 (0.786-0.845)	*P* = 0.0978	0.801 (0.770-0.830)	*P* = 0.2225	0.791 (0.759-0.820)	*P* = 0.0677	0.763 (0.730-0.794)	*P* = 0.0102
SAPSII∗RDW	0.833 (0.803-0.860)		0.813 (0.782-0.841)		0.805 (0.774-0.834)		0.782 (0.750-0.812)	
SOFA	0.787 (0.755-0.817)	*P* = 0.0025	0.740 (0.705-0.772)	*P* = 0.0006	0.726 (0.691-0.759)	*P* < 0.0001	0.671 (0.635-0.706)	*P* < 0.0001
SOFA∗RDW	0.802 (0.770-0.831)		0.757 (0.724-0.789)		0.743 (0.709-0.775)		0.692 (0.656-0.726)	
QSOFA	0.599 (0.561-0.635)	*P* < 0.0001	0.579 (0.541-0.616)	*P* < 0.0001	0.578 (0.541-0.615)	*P* < 0.0001	0.572 (0.534-0.608)	*P* < 0.0001
QSOFA∗RDW	0.677 (0.641-0.712)		0.664 (0.628-0.699)		0.659 (0.623-0.694)		0.648 (0.612-0.684)	
SIRS	0.576 (0.538-0.613)	*P* < 0.0001	0.577 (0.540-0.614)	*P* < 0.0001	0.567 (0.529-0.604)	*P* < 0.0001	0.546 (0.508-0.583)	*P* < 0.0001
SIRS∗RDW	0.644 (0.607-0.679)		0.646 (0.610-0.682)		0.634 (0.597-0.670)		0.605 (0.568-0.642)	
*Y*	0.851 (0.822-0.876)		0.821 (0.790-0.848)		0.810 (0.779-0.839)		0.788 (0.756-0.818)	

*Y* refers to the combination of RDW, SOFA, QSOFA, SIRS, LODS, and SAPSII in predicting mortality. The regression models are as follows: hospital mortality : *Y* = exp(−7.357 + 0.186∗RDW + 0.095∗SOFA + 0.178∗LODS − 0.354∗QSOFA + 0.048∗SAPSII − 0.166∗SIRS)/{1 + [exp(−7.357 + 0.186∗RDW + 0.095∗SOFA + 0.178∗LODS − 0.354∗QSOFA + 0.048∗SAPSII − 0.166∗SIRS)]}; 30-day mortality: *Y* = exp(−7.931 + 0.228∗RDW − 0.011∗SOFA + 0.152∗LODS − 0.423∗QSOFA + 0.057∗SAPSII − 0.053∗SIRS)/{1 + [exp(−7.931 + 0.228∗RDW − 0.011∗SOFA + 0.152∗LODS − 0.423∗QSOFA + 0.057∗SAPSII − 0.053∗SIRS)]}; 90-day mortality: *Y* = exp(−7.174 + 0.208∗RDW + 0.022∗SOFA + 0.115∗LODS − 0.400∗QSOFA + 0.065∗SAPSII − 0.082∗SIRS)/{1 + [exp(−7.174 + 0.208∗RDW + 0.022∗SOFA + 0.115∗LODS − 0.400∗QSOFA + 0.065∗SAPSII − 0.082∗SIRS)]}; and 1-year mortality: *Y* = exp(−6.576 + 0.226∗RDW − 0.048∗SOFA + 0.102∗LODS − 0.289∗QSOFA + 0.069∗SAPSII − 0.136∗SIRS)/{1 + [exp(−6.576 + 0.226∗RDW − 0.048∗SOFA + 0.102∗LODS − 0.289∗QSOFA + 0.069∗SAPSII − 0.136∗SIRS)]}.

**Table 3:** Area under the receiver operating curve of RDW for predicting in-hospital, 30-day, 90-day, and 1-year mortality in the sepsis and nonsepsis groups.

Group	Hospital mortality	30-day mortality	90-day mortality	1-year mortality
	AUC (95% CI)	AUC (95% CI)	AUC (95% CI)	AUC (95% CI)
Sepsis	0.688 (0.629, 0.748)	0.686 (0.622, 0.751)	0.693 (0.641, 0.746)	0.698 (0.647, 0.748)
No sepsis	0.606 (0.483, 0.729)	0.622 (0.489, 0.755)	0.595 (0.484, 0.705)	0.591 (0.511, 0.671)

**Table 4:** Relationship between RDW and hospital mortality in different models.

	Nonadjusted		Adjusted I		Adjusted II	
	OR (95% CI)	*P*	OR (95% CI)	*P*	OR (95% CI)	*P*
RDW	1.316 (1.202, 1.442)	<0.001	1.353 (1.230, 1.489)	<0.001	1.187 (1.049, 1.343)	0.007
RDW^Quintile^						
12.1-13.9%	1		1		1	
14.0-14.8%	2.600 (0.902, 7.494)	0.077	2.720 (0.933, 7.926)	0.067	2.787 (0.773, 10.053)	0.117
14.9-15.8%	5.899 (2.196, 15.849)	<0.001	6.133 (2.250, 16.718)	<0.001	4.902 (1.465, 16.408)	0.010
15.9-17.2%	5.353 (1.972, 14.530)	0.001	5.854 (2.125, 16.130)	0.001	3.972 (1.179, 13.385)	0.026
17.3-26.9%	11.152 (4.23, 29.399)	<0.001	13.638 (5.055, 36.792)	<0.001	6.119 (1.821, 20.565)	0.003
RDW *P* for trend	1.642 (1.391, 1.938)	<0.001	1.728 (1.452, 2.056)	<0.001	1.370 (1.103, 1.701)	0.004

CI = confidence interval; OR = odds ratio. Adjusted I for age, gender, and ethnicity. Adjusted II for age, ethnicity, time in hospital, first care unit, QSOFA, LODS, SAPSII, mean corpuscular volume, white blood count, infection, liver disease, blood loss anemia, deficiency anemias, and weight loss. Reference group: RDW level 12.1-13.9%.

**Table 5:** Relationship between RDW and 30-day, 90-day, and 1-year mortality in different models.

	Nonadjusted		Adjusted I		Adjusted II	
	HR (95% CI)	*P*	HR (95% CI)	*P*	HR (95% CI)	*P*
30-day mortality						
RDW	1.267 (1.185, 1.355)	<0.001	1.291 (1.207, 1.381)	<0.001	1.183 (1.080, 1.295)	<0.001
RDW^Quintile^						
12.1-13.9%	1		1		1	
14.0-14.8%	2.280 (0.876, 5.933)	0.091	2.310 (0.887, 6.014)	0.086	3.194 (1.107, 9.217)	0.032
14.9-15.8%	3.042 (1.207, 7.663)	0.018	2.960 (1.171, 7.483)	0.022	3.390 (1.223, 9.401)	0.019
15.9-17.2%	4.270 (1.745, 10.445)	0.001	4.465 (1.820, 10.954)	0.001	3.655 (1.364, 9.791)	0.010
17.3-26.9%	7.277 (3.060, 17.302)	<0.001	8.227 (3.443, 19.659)	<0.001	5.328 (1.990, 14.26)	0.001
RDW *P* for trend	1.551 (1.326, 1.813)	<0.001	1.610 (1.371, 1.890)	<0.001	1.337 (1.120, 1.597)	0.001
90-day mortality						
RDW	1.241 (1.173, 1.313)	<0.001	1.266 (1.196, 1.341)	<0.001	1.175 (1.089, 1.268)	<0.001
RDW^Quintile^						
12.1-13.9%	1		1		1	
14.0-14.8%	2.432 (1.12, 5.282)	0.025	2.458 (1.131, 5.340)	0.023	2.411 (1.064, 5.465)	0.035
14.9-15.8%	3.977 (1.907, 8.292)	<0.001	3.862 (1.847, 8.075)	<0.001	3.262 (1.503, 7.079)	0.003
15.9-17.2%	4.639 (2.239, 9.614)	<0.001	4.900 (2.359, 10.175)	<0.001	3.122 (1.454, 6.703)	0.004
17.3-26.9%	7.486 (3.681, 15.226)	<0.001	8.367 (4.099, 17.083)	<0.001	4.120 (1.919, 8.847)	<0.001
RDW *P* for trend	1.522 (1.345, 1.721)	<0.001	1.576 (1.389, 1.787)	<0.001	1.287 (1.123, 1.476)	<0.001
1-year mortality						
RDW	1.223 (1.164, 1.285)	<0.001	1.244 (1.184, 1.307)	<0.001	1.162 (1.089, 1.240)	<0.001
RDW^Quintile^						
12.1-13.9%	1		1		1	
14.0-14.8%	1.963 (1.093, 3.524)	0.024	1.971 (1.097, 3.539)	0.023	1.982 (1.081, 3.637)	0.027
14.9-15.8%	3.049 (1.751, 5.311)	<0.001	2.910 (1.667, 5.081)	<0.001	2.557 (1.435, 4.556)	0.001
15.9-17.2%	3.559 (2.055, 6.163)	<0.001	3.759 (2.165, 6.524)	<0.001	2.558 (1.444, 4.531)	0.001
17.3-26.9%	5.492 (3.216, 9.380)	<0.001	6.116 (3.568, 10.484)	<0.001	3.208 (1.812, 5.679)	<0.001
RDW *P* for trend	1.455 (1.314, 1.611)	<0.001	1.505 (1.356, 1.671)	<0.001	1.256 (1.123, 1.404)	<0.001

CI = confidence interval; HR = hazard ratio. Adjusted I for age, gender, and ethnicity. Adjusted II for age, ethnicity, time in hospital, first care unit, QSOFA, LODS, SAPSII, mean corpuscular volume, mean corpuscular hemoglobin, white blood count, red blood cell, blood urea nitrogen, hematocrit, creatinine, infection, liver disease, blood loss anemia, deficiency anemias, and weight loss. Reference group: RDW level 12.1-13.9%.

## Data Availability

The clinical data used to support the findings of this study were supplied by Monitoring in Intensive Care Database III (MIMIC-III). All data and materials are available at https://mimic.physionet.org/.

## Supplementary Materials

Supplementary Materials Figure S1: overall survival of different RDW groups. Kaplan-Meier curve of 180-day (A), 3-year (B), and 5-year (C) mortality. Table S1: comparison of short-term and long-term prognostic survival (%) of each group. Table S2: area under the receiver operating curve of RDW and scoring systems for predicting 180-day, 3-year, and 5-year mortality. Table S3: relationship between RDW and 180-day, 3-year, and 5-year mortality in different models. Table S4: subgroup analysis of the association between RDW and 1-year mortality.
