# Supplementary figures and images for: A Retrospective Cohort Study on the Association between Red Cell Distribution Width and All-Cause Mortality of Patients with Cholecystitis at ICU Admission
Source: Dis Markers. 2021 Oct 13;2021:9625220. doi: 10.1155/2021/9625220 (PMC8528576; doi:10.1155/2021/9625220)

RDW    + <14.0%    + 14.0–14.8%    + 14.9–15.8%    + 15.9–17.2%    + >17.2%

Survival probability

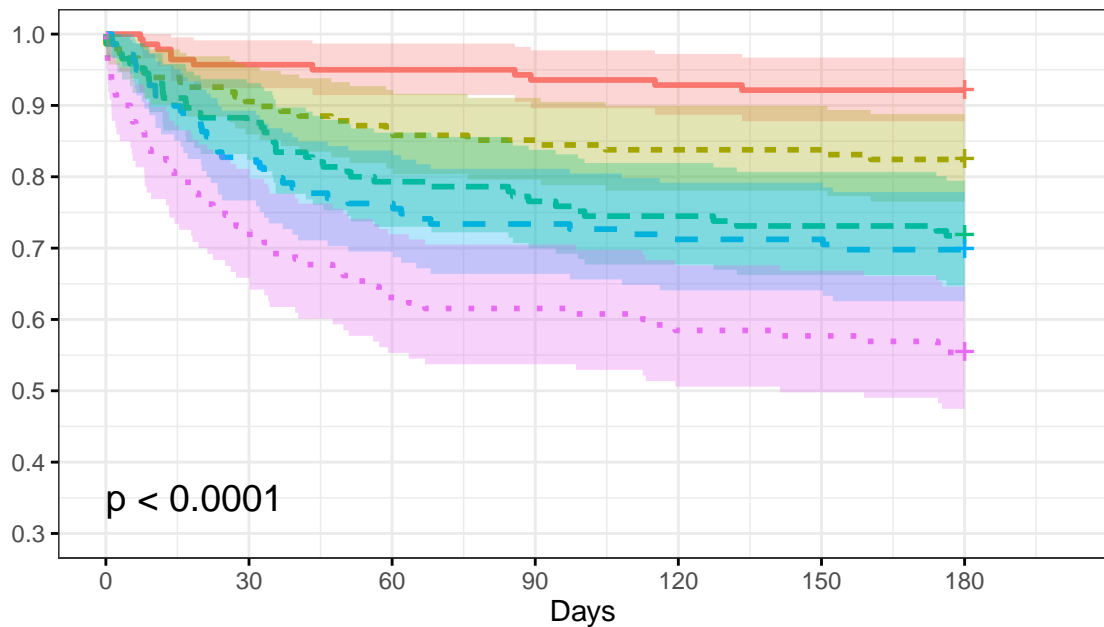

Number at risk

RDW

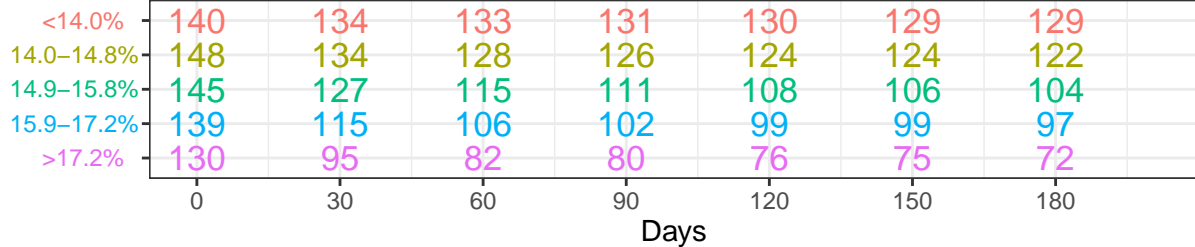

Supplement: Supplementary Materials — Figure S1: overall survival of different RDW groups. Kaplan-Meier curve of 180-day (A), 3-year (B), and 5-year (C) mortality. Table S1: comparison of short-term and long-term prognostic survival (%) of each group. Table S2: area under the receiver operating curve of RDW and scoring systems for predicting 180-day, 3-year, and 5-year mortality. Table S3: relationship between RDW and 180-day, 3-year, and 5-year mortality in different models. Table S4: subgroup analysis of the association between RDW and 1-year mortality. [file 9625220.f1.zip › Fig.S1.A.pdf]

RDW    + <14.0%    + 14.0–14.8%    + 14.9–15.8%    + 15.9–17.2%    + >17.2%

Survival probability

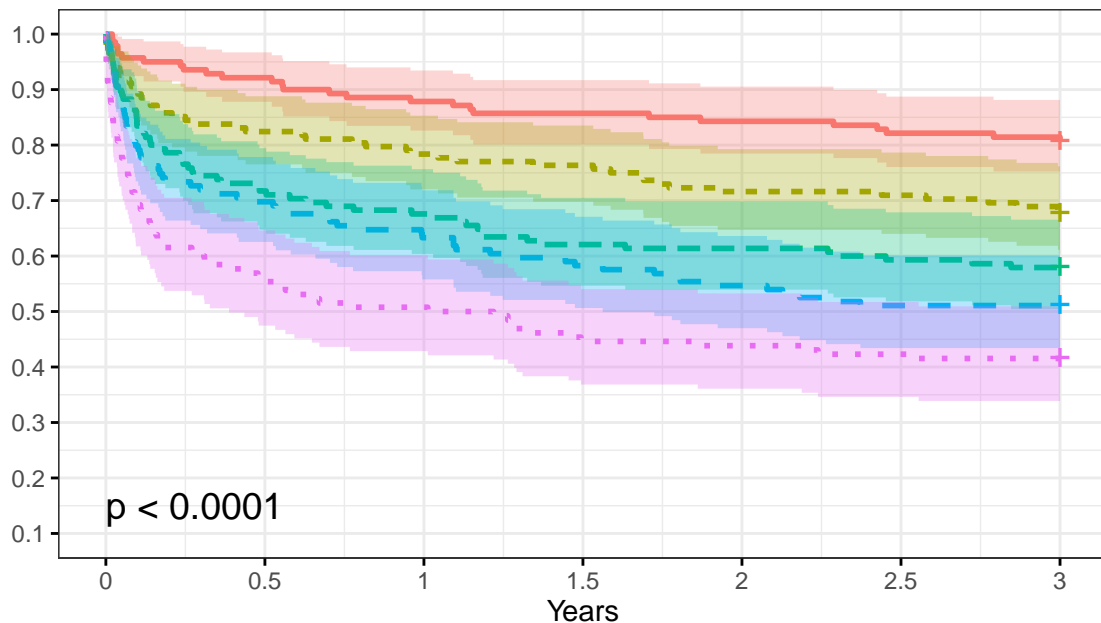

Number at risk

RDW

|            |     |     |     |     |     |     |     |
|------------|-----|-----|-----|-----|-----|-----|-----|
| <14.0%     | 140 | 129 | 123 | 120 | 118 | 115 | 114 |
| 14.0–14.8% | 148 | 122 | 116 | 113 | 106 | 105 | 101 |
| 14.9–15.8% | 145 | 104 | 98  | 90  | 89  | 86  | 84  |
| 15.9–17.2% | 139 | 97  | 88  | 81  | 76  | 71  | 71  |
| >17.2%     | 130 | 72  | 66  | 58  | 57  | 55  | 54  |
|            | 0   | 0.5 | 1   | 1.5 | 2   | 2.5 | 3   |

Years

Supplement: Supplementary Materials — Figure S1: overall survival of different RDW groups. Kaplan-Meier curve of 180-day (A), 3-year (B), and 5-year (C) mortality. Table S1: comparison of short-term and long-term prognostic survival (%) of each group. Table S2: area under the receiver operating curve of RDW and scoring systems for predicting 180-day, 3-year, and 5-year mortality. Table S3: relationship between RDW and 180-day, 3-year, and 5-year mortality in different models. Table S4: subgroup analysis of the association between RDW and 1-year mortality. [file 9625220.f1.zip › Fig.S1.B.pdf]
