# Supplementary material for: A Retrospective Cohort Study on the Association between Red Cell Distribution Width and All-Cause Mortality of Patients with Cholecystitis at ICU Admission
Source: Dis Markers. 2021 Oct 13;2021:9625220. doi: 10.1155/2021/9625220 (PMC8528576; doi:10.1155/2021/9625220)
Supplement: Supplementary Materials — Figure S1: overall survival of different RDW groups. Kaplan-Meier curve of 180-day (A), 3-year (B), and 5-year (C) mortality. Table S1: comparison of short-term and long-term prognostic survival (%) of each group. Table S2: area under the receiver operating curve of RDW and scoring systems for predicting 180-day, 3-year, and 5-year mortality. Table S3: relationship between RDW and 180-day, 3-year, and 5-year mortality in different models. Table S4: subgroup analysis of the association between RDW and 1-year mortality. [file Fig.S1.C.pdf › Fig.S1.C.pdf]

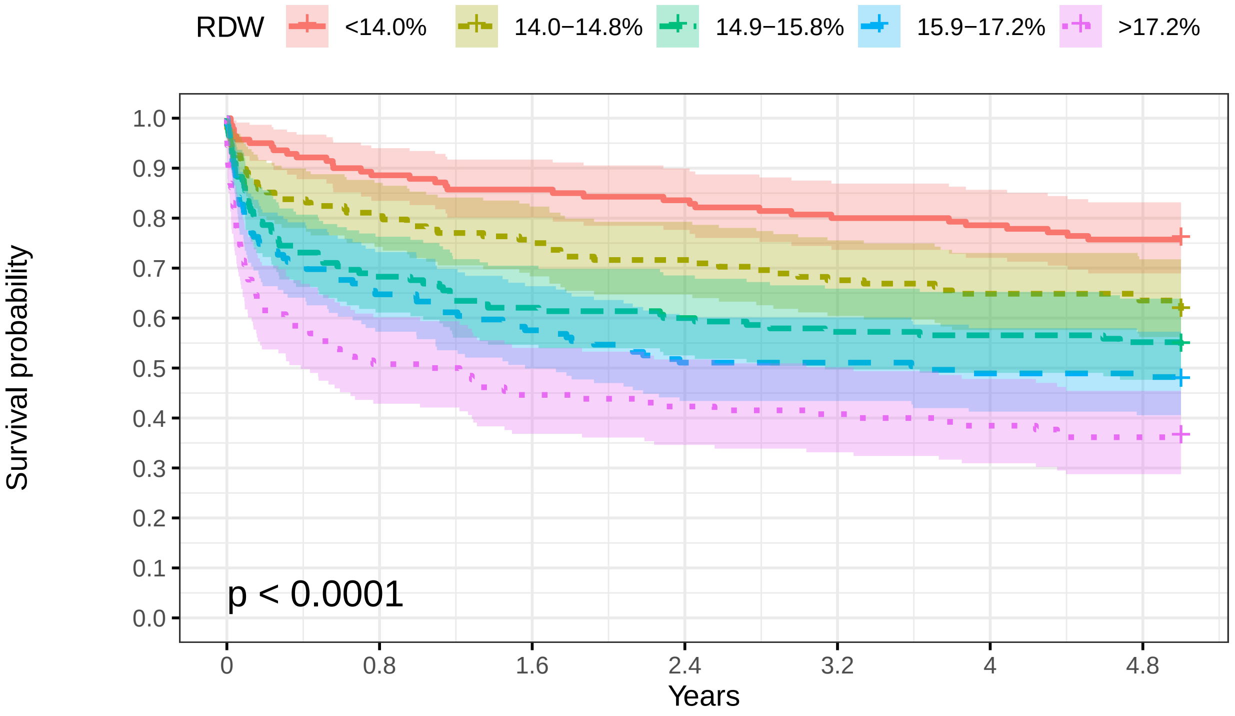

Number at risk

RDW	0	0.8	1.6	2.4	3.2	4	4.8
<14.0%	140	124	120	117	112	110	106
14.0−14.8%	148	119	111	106	100	96	94
14.9−15.8%	145	99	90	87	83	82	80
15.9−17.2%	139	90	80	71	71	68	67
>17.2%	130	66	58	55	53	50	47

Years
