# Supplementary material for: A Retrospective Cohort Study on the Association between Red Cell Distribution Width and All-Cause Mortality of Patients with Cholecystitis at ICU Admission
Source: Dis Markers. 2021 Oct 13;2021:9625220. doi: 10.1155/2021/9625220 (PMC8528576; doi:10.1155/2021/9625220)
Supplement: Supplementary Materials — Figure S1: overall survival of different RDW groups. Kaplan-Meier curve of 180-day (A), 3-year (B), and 5-year (C) mortality. Table S1: comparison of short-term and long-term prognostic survival (%) of each group. Table S2: area under the receiver operating curve of RDW and scoring systems for predicting 180-day, 3-year, and 5-year mortality. Table S3: relationship between RDW and 180-day, 3-year, and 5-year mortality in different models. Table S4: subgroup analysis of the association between RDW and 1-year mortality. [file 9625220.f1.zip › Table S1.doc]

**Table S1.** Comparison of short-term and long-term prognostic survival (%) of each group.

|  | ≤13.9% | 14.0-14.8% | 14.9-15.8% | 15.9-17.2% | ≥17.3% | p value |
| --- | --- | --- | --- | --- | --- | --- |
| 30-day | 95.70% | 90.50% | 87.60% | 82.70% | 73.10% | <0.001 |
| 90-day | 93.60% | 85.10% | 76.60% | 73.40% | 61.50% | <0.001 |
| 180-day | 92.10% | 82.40% | 71.70% | 69.80% | 55.40% | <0.001 |
| 1-year | 87.90% | 77.70% | 67.60% | 63.30% | 50.80% | <0.001 |
| 3-year | 80.70% | 67.60% | 57.90% | 51.10% | 41.50% | <0.001 |
| 5-year | 75.70% | 61.50% | 54.50% | 47.50% | 36.20% | <0.001 |
