# Supplementary material for: A Retrospective Cohort Study on the Association between Red Cell Distribution Width and All-Cause Mortality of Patients with Cholecystitis at ICU Admission
Source: Dis Markers. 2021 Oct 13;2021:9625220. doi: 10.1155/2021/9625220 (PMC8528576; doi:10.1155/2021/9625220)
Supplement: Supplementary Materials — Figure S1: overall survival of different RDW groups. Kaplan-Meier curve of 180-day (A), 3-year (B), and 5-year (C) mortality. Table S1: comparison of short-term and long-term prognostic survival (%) of each group. Table S2: area under the receiver operating curve of RDW and scoring systems for predicting 180-day, 3-year, and 5-year mortality. Table S3: relationship between RDW and 180-day, 3-year, and 5-year mortality in different models. Table S4: subgroup analysis of the association between RDW and 1-year mortality. [file 9625220.f1.zip › Table S2.doc]

**Table S2.** Area under receiver operating curve of RDW and scoring systems for predicting180-day, 3-year and 5-year mortality.

|  | 180-day mortality | |  | 3-year mortality | |  | 5-year mortality | |
| --- | --- | --- | --- | --- | --- | --- | --- | --- |
|  | AUC (95% CI) | p |  | AUC (95% CI) | p |  | AUC (95% CI) | p |
| RDW | 0.687（0.651 - 0.721） |  |  | 0.662（0.626 - 0.697） |  |  | 0.652（0.615 - 0.687） |  |
| LODS | 0.751（0.717 - 0.783） | P = 0.0004 |  | 0.689（0.654 - 0.724） | P < 0.0001 |  | 0.685（0.649 - 0.719） | P < 0.0001 |
| LODS*RDW | 0.765（0.732 - 0.796） |  |  | 0.706（0.671 - 0.740） |  |  | 0.701（0.665 - 0.734） |  |
| SAPSII | 0.794（0.762 - 0.823） | P = 0.0264 |  | 0.733（0.698 - 0.765） | P = 0.0008 |  | 0.725（0.690 - 0.757） | P = 0.0015 |
| SAPSII*RDW | 0.81（0.779 - 0.838） |  |  | 0.755（0.722 - 0.787） |  |  | 0.746（0.712 - 0.777） |  |
| SOFA | 0.706（0.671 - 0.739） | P < 0.0001 |  | 0.645（0.609 - 0.681） | P < 0.0001 |  | 0.645（0.608 - 0.680） | P < 0.0001 |
| SOFA*RDW | 0.726（0.691 - 0.759） |  |  | 0.667（0.630 - 0.701） |  |  | 0.664（0.628 - 0.699） |  |
| QSOFA | 0.590（0.552 - 0.626） | P < 0.0001 |  | 0.583（0.545 - 0.619） | P < 0.0001 |  | 0.584（0.546 - 0.620） | P < 0.0001 |
| QSOFA*RDW | 0.670（0.634 - 0.705） |  |  | 0.656（0.620 - 0.691） |  |  | 0.652（0.616 - 0.687） |  |
| SIRS | 0.556（0.518 - 0.593） | P < 0.0001 |  | 0.535（0.497 - 0.572） | P < 0.0001 |  | 0.520（0.482 - 0.558） | P < 0.0001 |
| SIRS*RDW | 0.623（0.586 - 0.659） |  |  | 0.591（0.554 - 0.628） |  |  | 0.573（0.536 - 0.610） |  |
