# Supplementary material for: A Retrospective Cohort Study on the Association between Red Cell Distribution Width and All-Cause Mortality of Patients with Cholecystitis at ICU Admission
Source: Dis Markers. 2021 Oct 13;2021:9625220. doi: 10.1155/2021/9625220 (PMC8528576; doi:10.1155/2021/9625220)
Supplement: Supplementary Materials — Figure S1: overall survival of different RDW groups. Kaplan-Meier curve of 180-day (A), 3-year (B), and 5-year (C) mortality. Table S1: comparison of short-term and long-term prognostic survival (%) of each group. Table S2: area under the receiver operating curve of RDW and scoring systems for predicting 180-day, 3-year, and 5-year mortality. Table S3: relationship between RDW and 180-day, 3-year, and 5-year mortality in different models. Table S4: subgroup analysis of the association between RDW and 1-year mortality. [file 9625220.f1.zip › Table S3.doc]

**Table S3.** Relationship between RDW and 180-day, 3-year and 5-year mortality in different models.

|  | Non-adjusted |  |  | Adjusted I |  |  | Adjusted II |  |
| --- | --- | --- | --- | --- | --- | --- | --- | --- |
|  | HR (95%CI) | P |  | HR (95%CI) | P |  | HR (95%CI) | P |
| 180-day mortality |  |  |  |  |  |  |  |  |
| RDW | 1.238 (1.175, 1.305) | <0.001 |  | 1.265 (1.199, 1.334) | <0.001 |  | 1.180 (1.099, 1.267) | <0.001 |
| RDW ^Quintile^ |  |  |  |  |  |  |  |  |
| 12.1-13.9% | 1 |  |  | 1 |  |  | 1 |  |
| 14.0-14.8% | 2.372 (1.172, 4.801) | 0.016 |  | 2.398 (1.185, 4.856) | 0.015 |  | 2.311 (1.108, 4.819) | 0.025 |
| 14.9-15.8% | 4.010 (2.061, 7.802) | <0.001 |  | 3.879 (1.989, 7.567) | <0.001 |  | 3.240 (1.617, 6.491) | 0.001 |
| 15.9-17.2% | 4.410 (2.270, 8.566) | <0.001 |  | 4.717 (2.423, 9.183) | <0.001 |  | 3.066 (1.537, 6.116) | 0.001 |
| 17.3-26.9% | 7.355 (3.859, 14.017) | <0.001 |  | 8.303 (4.341, 15.881) | <0.001 |  | 4.072 (2.044, 8.115) | <0.001 |
| RDW P for trend | 1.516 (1.353, 1.697) | <0.001 |  | 1.575 (1.403, 1.769) | <0.001 |  | 1.294 (1.141, 1.467) | <0.001 |
| 3-year mortality |  |  |  |  |  |  |  |  |
| RDW | 1.207 (1.155, 1.263) | <0.001 |  | 1.235 (1.181, 1.292) | <0.001 |  | 1.157 (1.092, 1.226) | <0.001 |
| RDW ^Quintile^ |  |  |  |  |  |  |  |  |
| 12.1-13.9% | 1 |  |  | 1 |  |  | 1 |  |
| 14.0-14.8% | 1.833 (1.144, 2.937) | 0.012 |  | 1.842 (1.149, 2.954) | 0.011 |  | 1.764 (1.086, 2.864) | 0.022 |
| 14.9-15.8% | 2.611 (1.660, 4.109) | <0.001 |  | 2.496 (1.583, 3.937) | <0.001 |  | 2.196 (1.373, 3.511) | 0.001 |
| 15.9-17.2% | 3.180 (2.035, 4.967) | <0.001 |  | 3.389 (2.164, 5.307) | <0.001 |  | 2.366 (1.489, 3.761) | <0.001 |
| 17.3-26.9% | 4.450 (2.867, 6.908) | <0.001 |  | 5.085 (3.262, 7.927) | <0.001 |  | 2.847 (1.773, 4.573) | <0.001 |
| RDW P for trend | 1.398 (1.281, 1.525) | <0.001 |  | 1.454 (1.329, 1.590) | <0.001 |  | 1.247 (1.131, 1.375) | <0.001 |
| 5-year mortality |  |  |  |  |  |  |  |  |
| RDW | 1.194 (1.144, 1.247) | <0.001 |  | 1.222 (1.169, 1.276) | <0.001 |  | 1.148 (1.085, 1.214) | <0.001 |
| RDW ^Quintile^ |  |  |  |  |  |  |  |  |
| 12.1-13.9% | 1 |  |  | 1 |  |  | 1 |  |
| 14.0-14.8% | 1.747 (1.142, 2.671) | 0.010 |  | 1.735 (1.134, 2.655) | 0.011 |  | 1.64 (1.061, 2.536) | 0.026 |
| 14.9-15.8% | 2.282 (1.508, 3.452) | <0.001 |  | 2.153 (1.42, 3.264) | <0.001 |  | 1.946 (1.269, 2.984) | 0.002 |
| 15.9-17.2% | 2.781 (1.851, 4.18) | <0.001 |  | 2.944 (1.955, 4.435) | <0.001 |  | 2.111 (1.382, 3.223) | 0.001 |
| 17.3-26.9% | 3.998 (2.681, 5.963) | <0.001 |  | 4.516 (3.015, 6.765) | <0.001 |  | 2.62 (1.699, 4.042) | <0.001 |
| RDW P for trend | 1.366 (1.259, 1.483) | <0.001 |  | 1.418 (1.303, 1.542) | <0.001 |  | 1.23 (1.122, 1.348) | <0.001 |
| CI=confidence interval HR=hazard ratio |  |  |  |  |  |  |  |  |
| Adjusted I for age, gender, ethnicity. | | | | | | |  |  |
| Adjusted II for age, ethnicity, time in hospital, first care unit, QSOFA, LODS, SAPS II, mean corpuscular volume, mean corpuscular hemoglobin, white blood count, red blood cell, blood urea nitrogen, hematocrit, creatinine, infection, liver disease, blood loss anemia, deficiency anemias, weight loss. | | | | | | | | |
| Reference group: RDW level 12.1-13.9%. | | | | | | | | |
