# Supplementary material for: A Retrospective Cohort Study on the Association between Red Cell Distribution Width and All-Cause Mortality of Patients with Cholecystitis at ICU Admission
Source: Dis Markers. 2021 Oct 13;2021:9625220. doi: 10.1155/2021/9625220 (PMC8528576; doi:10.1155/2021/9625220)
Supplement: Supplementary Materials — Figure S1: overall survival of different RDW groups. Kaplan-Meier curve of 180-day (A), 3-year (B), and 5-year (C) mortality. Table S1: comparison of short-term and long-term prognostic survival (%) of each group. Table S2: area under the receiver operating curve of RDW and scoring systems for predicting 180-day, 3-year, and 5-year mortality. Table S3: relationship between RDW and 180-day, 3-year, and 5-year mortality in different models. Table S4: subgroup analysis of the association between RDW and 1-year mortality. [file 9625220.f1.zip › Table S4.doc]

**Table S4. Subgroup analysis of the association between RDW and 1-year mortality.**

|  | N | HR (95%CI) | P for interaction |
| --- | --- | --- | --- |
| Age |  |  | <0.001 |
| 23-69 | 341 | 1.215 (1.129, 1.308) |  |
| 70-92 | 361 | 1.266 (1.183, 1.356) |  |
| Sex |  |  | 0.047 |
| Female | 304 | 1.288 (1.188, 1.396) |  |
| Male | 398 | 1.186 (1.112, 1.265) |  |
| SOFA |  |  | <0.001 |
| 0-3.0 | 234 | 1.100 (0.971, 1.246) |  |
| 4.0-7.0 | 297 | 1.286 (1.177, 1.406) |  |
| 8.0-21.0 | 171 | 1.152 (1.062, 1.249) |  |
| SAPSII |  |  | <0.001 |
| 0-33.0 | 244 | 1.192 (1.052, 1.349) |  |
| 34.0-44.0 | 238 | 1.132 (1.017, 1.261) |  |
| 45.0-107.0 | 220 | 1.236 (1.148, 1.331) |  |
| QSOFA, n (%) |  |  | <0.001 |
| 0 | 27 | 0.906 (0.574, 1.430) |  |
| 1 | 204 | 1.102 (0.983, 1.234) |  |
| 2 | 382 | 1.318 (1.235, 1.407) |  |
| 3 | 89 | 1.144 (1.008, 1.299) |  |
| SIRS, n (%) |  |  | <0.001 |
| 0 | 5 | 0.861 (0.394, 1.880) |  |
| 1 | 42 | 1.280 (1.006, 1.627) |  |
| 2 | 157 | 1.274 (1.099, 1.476) |  |
| 3 | 259 | 1.200 (1.108, 1.301) |  |
| 4 | 239 | 1.223 (1.132, 1.321) |  |
| LODS |  |  | <0.001 |
| 0-3.0 | 289 | 1.171 (1.044, 1.312) |  |
| 4.0-5.0 | 182 | 1.131 (1.007, 1.269) |  |
| 6.0-18.0 | 231 | 1.226 (1.138, 1.321) |  |
| First care unit |  |  | 0.912 |
| MICU | 259 | 1.274 (1.191, 1.363) |  |
| CCU | 36 | 1.435 (1.195, 1.724) |  |
| SICU | 257 | 1.151 (1.006, 1.318) |  |
| CSRU | 56 | 1.092 (0.894, 1.335) |  |
| TSICU | 94 | 1.060 (0.908, 1.237) |  |
| Admission type |  |  | 0.982 |
| Emergency | 544 | 1.250 (1.181, 1.323) |  |
| Elective | 137 | 1.076 (0.931, 1.244) |  |
| Urgent | 21 | 1.607 (0.925, 2.792) |  |
| Ethnicity, n (%) |  |  | 0.001 |
| White | 535 | 1.226 (1.158, 1.298) |  |
| Black | 58 | 1.226 (1.064, 1.413) |  |
| Hispanic | 21 | 0.964 (0.577, 1.610) |  |
| Others | 88 | 1.272 (1.097, 1.474) |  |
| Infection |  |  | <0.001 |
| 0 | 227 | 1.187 (1.042, 1.351) |  |
| 1 | 475 | 1.214 (1.151, 1.281) |  |
| Sepsis |  |  | <0.001 |
| 0 | 268 | 1.162 (1.026, 1.316) |  |
| 1 | 434 | 1.215 (1.152, 1.283) |  |
| Congestive heart failure |  |  | <0.001 |
| 0 | 485 | 1.18 (1.105, 1.259) |  |
| 1 | 217 | 1.307 (1.20, 1.424) |  |
| Cardiac arrhythmias |  |  | <0.001 |
| 0 | 418 | 1.149 (1.069, 1.235) |  |
| 1 | 284 | 1.34 (1.242, 1.446) |  |
| Hypertension |  |  | 0.171 |
| 0 | 294 | 1.189 (1.106, 1.277) |  |
| 1 | 408 | 1.266 (1.180, 1.359) |  |
| Diabetes |  |  | 0.306 |
| 0 | 484 | 1.203 (1.136, 1.275) |  |
| 1 | 218 | 1.29 (1.167, 1.426) |  |
| Liver disease |  |  | <0.001 |
| 0 | 579 | 1.242 (1.175, 1.312) |  |
| 1 | 123 | 1.087 (0.964, 1.225) |  |
| Blood loss anemia |  |  | 0.711 |
| 0 | 683 | 1.222 (1.162, 1.284) |  |
| 1 | 19 | 1.282 (0.863, 1.904) |  |
| Deficiency anemias |  |  | 0.507 |
| 0 | 684 | 1.228 (1.169, 1.290) |  |
| 1 | 18 | 0.842 (0.529, 1.340) |  |
| Fluid electrolyte |  |  | <0.001 |
| 0 | 465 | 1.209 (1.134, 1.290) |  |
| 1 | 237 | 1.248 (1.153, 1.352) |  |
| Metastatic cancer |  |  | <0.001 |
| 0 | 646 | 1.219 (1.156, 1.285) |  |
| 1 | 56 | 1.323 (1.086, 1.612) |  |
| Solid tumor |  |  | 0.824 |
| 0 | 680 | 1.239 (1.177, 1.303) |  |
| 1 | 22 | 0.995 (0.737, 1.344) |  |
| Mean corpuscular volume |  |  | <0.001 |
| 65.0-88.0 | 246 | 1.151 (1.040, 1.275) |  |
| 89.0-93.0 | 228 | 1.285 (1.158, 1.427) |  |
| 94.0-121.0 | 228 | 1.235 (1.144, 1.334) |  |
| Mean corpuscular hemoglobin |  |  | <0.001 |
| 20.3-29.8 | 238 | 1.18 (1.080, 1.289) |  |
| 29.9-31.6 | 241 | 1.262 (1.156, 1.379) |  |
| 31.7-40.2 | 223 | 1.254 (1.148, 1.370) |  |
| Red blood cell |  |  | 0.423 |
| 2.17-3.49 | 234 | 1.236 (1.145, 1.334) |  |
| 3.50-3.95 | 238 | 1.152 (1.051, 1.262) |  |
| 3.96-5.82 | 230 | 1.314 (1.148, 1.503) |  |
| White blood count |  |  | 0.001 |
| 0.1-11.6 | 236 | 1.209 (1.118, 1.307) |  |
| 11.7-17.2 | 233 | 1.159 (1.055, 1.274) |  |
| 17.3-68.5 | 233 | 1.37 (1.239, 1.516) |  |
| Blood urea nitrogen |  |  | <0.001 |
| 3.0-18.0 | 260 | 1.07 (0.941, 1.217) |  |
| 19.0-29.0 | 220 | 1.219 (1.097, 1.356) |  |
| 30.0-251.0 | 221 | 1.25 (1.161, 1.346) |  |
| Hematocrit |  |  | 0.051 |
| 21.8-32.0 | 241 | 1.191 (1.108, 1.279) |  |
| 32.1-36.1 | 230 | 1.354 (1.213, 1.512) |  |
| 36.2-59.0 | 231 | 1.285 (1.132, 1.458) |  |
| Creatinine |  |  | <0.001 |
| 0.1-0.9 | 254 | 1.067 (0.935, 1.219) |  |
| 1.0-1.5 | 221 | 1.247 (1.122, 1.387) |  |
| 1.6-12.0 | 226 | 1.236 (1.151, 1.327) |  |
